# Overexpression of the Endothelial Protein C Receptor Is Detrimental during Pneumonia-Derived Gram-negative Sepsis (Melioidosis)

**DOI:** 10.1371/journal.pntd.0002306

**Published:** 2013-07-11

**Authors:** Liesbeth M. Kager, Marcel Schouten, W. Joost Wiersinga, J. Daan de Boer, Lionel C. W. Lattenist, Joris J. T. H. Roelofs, Joost C. M. Meijers, Marcel Levi, Arjen M. Dondorp, Charles T. Esmon, Cornelis van 't Veer, Tom van der Poll

**Affiliations:** 1 Center for Infection and Immunity Amsterdam (CINIMA), Academic Medical Center, University of Amsterdam, Amsterdam, The Netherlands; 2 Center for Experimental and Molecular Medicine (CEMM), Academic Medical Center, University of Amsterdam, Amsterdam, The Netherlands; 3 Department of Internal Medicine, Academic Medical Center, University of Amsterdam, Amsterdam, The Netherlands; 4 Department of Pathology, Academic Medical Center, University of Amsterdam, Amsterdam, The Netherlands; 5 Department of Experimental Vascular Medicine, Academic Medical Center, University of Amsterdam, Amsterdam, The Netherlands; 6 Mahidol-Oxford Tropical Medicine Research Unit, Faculty of Tropical Medicine, Mahidol University, Bangkok, Thailand; 7 Centre for Tropical Medicine, Nuffield Department of Clinical Medicine, John Radcliffe Hospital, Oxford, United Kingdom; 8 Oklahoma Medical Research Foundation, Howard Hughes Medical Institute, Oklahoma City, Oklahoma, United States of America; University of California San Diego School of Medicine, United States of America

## Abstract

**Background:**

The endothelial protein C receptor (EPCR) enhances anticoagulation by accelerating activation of protein C to activated protein C (APC) and mediates anti-inflammatory effects by facilitating APC-mediated signaling via protease activated receptor-1. We studied the role of EPCR in the host response during pneumonia-derived sepsis instigated by *Burkholderia (B.) pseudomallei*, the causative agent of melioidosis, a common form of community-acquired Gram-negative (pneumo)sepsis in South-East Asia.

**Methodology/Principal Findings:**

Soluble EPCR was measured in plasma of patients with septic culture-proven melioidosis and healthy controls. Experimental melioidosis was induced by intranasal inoculation of *B. pseudomallei* in wild-type (WT) mice and mice with either EPCR-overexpression (Tie2-EPCR) or EPCR-deficiency (EPCR^−/−^). Mice were sacrificed after 24, 48 or 72 hours. Organs and plasma were harvested to measure colony forming units, cellular influxes, cytokine levels and coagulation parameters. Plasma EPCR-levels were higher in melioidosis patients than in healthy controls and associated with an increased mortality. Tie2-EPCR mice demonstrated enhanced bacterial growth and dissemination to distant organs during experimental melioidosis, accompanied by increased lung damage, neutrophil influx and cytokine production, and attenuated coagulation activation. EPCR^−/−^ mice had an unremarkable response to *B. pseudomallei* infection as compared to WT mice, except for a difference in coagulation activation in plasma.

**Conclusion/Significance:**

Increased EPCR-levels correlate with accelerated mortality in patients with melioidosis. In mice, transgenic overexpression of EPCR aggravates outcome during Gram-negative pneumonia-derived sepsis caused by *B. pseudomallei*, while endogenous EPCR does not impact on the host response. These results add to a better understanding of the regulation of coagulation during severe (pneumo)sepsis.

## Author Summary

During pneumonia and sepsis a procoagulant state is observed, with activation of coagulation and downregulation of anticoagulant pathways, both closely interrelated with inflammation. The protein C (PC) system is an important anticoagulant pathway implicated in the pathogenesis of sepsis. The rate of conversion of the zymogen PC into the active end product of the PC system, activated PC, is enhanced when PC binds to the endothelial protein C receptor (EPCR). We studied the role of EPCR in the host response during pneumonia-derived sepsis instigated by *Burkholderia (B.) pseudomallei*, the causative agent of melioidosis, a common form of community-acquired Gram-negative (pneumo)sepsis in South-East Asia and a serious potential bioterrorism threat agent. Plasma EPCR-levels measured in culture-proven melioidosis patients were higher than in healthy controls and were associated with an increased mortality. Furthermore, mice overexpressing EPCR demonstrated enhanced bacterial growth and dissemination to distant organs during experimental melioidosis, accompanied by increased lung damage, neutrophil influx and cytokine production and attenuated coagulation activation, while endogenous EPCR does not impact on the host response. These data point to a possible role for EPCR in the host defense against *B. pseudomallei* and may be of value in the development of therapeutic strategies against this dangerous pathogen.

## Introduction

Melioidosis, caused by the soil-dwelling Gram-negative bacterium *Burkholderia* (*B.*) *pseudomallei*, is associated with pneumonia and bacterial dissemination to distant sites and is an important cause of community-acquired sepsis in South-East Asia [1]–[5]. The mortality of the primary disease is high and varies from 20 to 50% [1], [2]. Treatment of patients with melioidosis is often difficult with slow fever clearance-times, a need for prolonged antibiotic therapy and a high relapse rate if therapy is not fully completed. Additionally, *B. pseudomallei* is recently classified as a ‘Tier 1’ disease agent considered to be an exceptional threat to security [6].

During pneumonia and sepsis a procoagulant state is elicited in the host, with activation of coagulation and downregulation of anticoagulant pathways [7], [8]. The protein C (PC) system is an important anticoagulant pathway implicated in the pathogenesis of sepsis [9], [10]. PC is rapidly converted into activated protein C (APC) by the transmembrane receptor thrombomodulin, once activated by thrombin. The rate of this conversion of PC into APC is dramatically enhanced when PC binds to the endothelial protein C receptor (EPCR) [11], [12]. Besides its well-known anticoagulant functions, through proteolytical degradation of coagulation factors Va and VIIIa, APC can exert anti-inflammatory, anti-apoptotic and barrier protective signals in endothelial cells via protease activated receptor (PAR)1 by a mechanism that requires binding of APC to EPCR. Recent evidence demonstrates that EPCR also serves as a binding site for FVII(a) [13]–[15], facilitating PAR1 mediated cell signalling and barrier protection [15].

EPCR was originally identified as a transmembrane endothelial cell receptor, but has since also been detected on a number of other cell types including neutrophils, monocytes, eosinophils, keratinocytes, vascular smooth muscle cells and renal tubular epithelial cells [12], [16], [17]. The extracellular domain of membrane-bound EPCR (mEPCR) can be shed resulting in soluble EPCR (sEPCR) [18], which retains its affinity to bind PC and APC [19]. Previous studies have shown evidence for a role for EPCR during sepsis. Inhibition of EPCR-binding of PC and APC with EPCR-blocking antibodies was found to exacerbate the septic response in baboons [20], [21]. In addition, conditional EPCR-gene deletion, resulting in absent mEPCR on vascular membranes, exaggerated host responses to lipopolysaccharide (LPS), reflected by enhanced thrombin and cytokine generation, increased neutrophil sequestration in the lung and a higher mortality rate, and this was primarily due to deficiency of EPCR on non-hematopoietic cells [22]. In contrast, mice overexpressing EPCR, that as a consequence thereof generated more APC in response to thrombin, were protected against LPS-challenge [23]. Recently, it was demonstrated that EPCR is expressed by mouse CD8+ dendritic cells and that these cells are required for APC to provide protection against the lethality of sepsis [24]. Contrary to the well-known antithrombotic and anti-inflammatory effects of mEPCR, the functions of sEPCR are less clear. Increased [25]–[27], but also unchanged [28] or decreased [29] levels of sEPCR have been reported in patients with systemic inflammatory diseases and high sEPCR levels seem to be correlated with a poor outcome in severe sepsis [27].

In patients with melioidosis activation of pro-coagulant pathways has been observed, with concurrent impairment of anti-coagulant mechanisms [30]–[32]. In particular, levels of thrombin-antithrombin complexes (TATc), indicating coagulation activation, are significantly elevated, while levels of PC are decreased in these patients [32], which was associated with a higher mortality [30]. To the best of our knowledge, no data exist about the role of EPCR during melioidosis. In this study we measured sEPCR levels in melioidosis patients and investigated the effects of overexpression and absence of EPCR during this disease by using our established mouse model, in which *B. pseudomallei* is administered via the airways, mimicking pneumonia and septic disease [33]–[35].

## Methods

### Patients

Thirty-four patients with sepsis caused by *B. pseudomallei* (mean age 52 y, range 18–86 y; 50% male) and 32 healthy controls from the same area (mean age 41 y, range 21–59 y; 71% male) were studied. Study design and subjects have been described in detail [30], [35]. All subjects were recruited prospectively at Sapprasithiprasong Hospital, Ubon Ratchathani, Thailand. Sepsis due to melioidosis was defined as culture-positivity for *B. pseudomallei* from any clinical sample plus a systemic inflammatory response syndrome [36]. Blood samples were taken at admission. When possible, an additional blood sample was drawn from patients who recovered after 2 weeks of treatment. *B. pseudomallei* was cultured from body material from all patients: blood cultures were positive for *B.pseudomallei* in 21 patients (61.7%), throat swab or tracheal suction in 13 patients (38.0%), sputum in seven patients (21.0%), pus from abscesses in seven patients (21.0%), and urine in five patients (14.7%). No co-infections were present and none of the subjects were tested positive for HIV [35].

### Mice

Pathogen-free 10-week old male wild type (WT) C57BL/6 mice were purchased from Charles River (Maastricht, The Netherlands). Mice overexpressing the endothelial protein C receptor (Tie2-EPCR) and mice conditionally knockout for the EPCR-gene (EPCR^−/−^) were generated as described [23], [37]. Overexpression of EPCR was achieved by placing the expression of the EPCR-gene under a Tie-2 promoter [23]. EPCR overexpression enhances PC activation on endothelial cells eightfold [23]. Meox2^+/*cre*^ *Procr^−/−^* mice (abbreviated as EPCR^−/−^ mice) were generated as described [37]. Briefly, via a conditional knockout system (*cre-Lox*) the EPCR-gene was deleted in embryos while the necessary EPCR-expression on placental giant trophoblasts was left intact, enabling the embryos to be carried to term and to develop normally [37]. Overexpression or homozygous deficiency for EPCR was checked by PCR analysis of each mouse used in the experiment. Both strains were backcrossed on a C57BL/6 background for at least 6 times.

### Ethics statement

The patient study was approved by the Ministry of Public Health, Royal Government of Thailand and the Oxford Tropical Research Ethics Committee, University of Oxford, Oxford, UK. Written informed consent was obtained from all subjects before the study. The animal study was carried out under the guidance of the Animal Research Institute of the Academical Medical Center in Amsterdam (ARIA). All animals were maintained at the animal care facility of the Academic Medical Center (University of Amsterdam), with free access to food and water, according to National Guidelines for the Care and Use of Laboratory Animals, which are based on the National Experiments on Animals Act (Wet op de Dierproeven (WOD)) and the Experiments on Animals Decree (Dierproevenbesluit), under the jurisdiction of the Ministry of Public Health, Welfare and Sports, the Netherlands. The Committee of Animal Care and Use (Dier Experimenten Commissie, DEC) of the University of Amsterdam approved all experiments (Permit numbers DIX100121-101237 and −101679)

### Experimental infection and determination of bacterial growth

Pneumonia was induced by intranasal inoculation with *B. pseudomallei* strain 1026b (300 colony forming units (CFU) in 50 µL NaCl 0.9% as previously described [33]–[35]. Mice were sacrificed after 24, 48 or 72 hours and survival studies were performed. Sample harvesting, processing and determination of bacterial growth were done as described [33]–[35]. Briefly, mice were sacrificed under intraperitoneal anaesthesia containing ketamin (Eurovet Animal Health, Bladel, The Netherlands) and medetomidin (Pfizer Animal Health Care, Capelle aan den IJssel, The Netherlands). Blood was drawn into syringes containing sodium citrate (4∶1 vol/vol). Lungs and liver were harvested and homogenized at 4°C in 4 volumes of sterile saline using a tissue homogenizer (Biospec Products, Bartlesville, OK). CFU were determined from serial dilutions of organ homogenates and blood plated on blood agar plates and incubated at 37°C 5% CO_2_ for 20 h before colonies were counted.

### Assays

In patients, soluble EPCR (sEPCR) was measured with a commercially available ELISA kit (R&D systems, Minneapolis, MN) according to the manufacturers' instructions. In mice, samples were processed for ELISA measurements as described [33]–[35]. In brief, lung homogenates were diluted 1∶2 in lysis buffer containing 300 mM NaCl, 30 mM Tris, 2 mM MgCl_2_, 2 mMCaCl_2_, 1% Triton X-100 and Pepstatin A, Leupeptin and Aprotinin (all 20 ng/mL, pH 7.4), incubated at 4°C for 30 min and centrifuged at 1730× *g* at 4°C for 10 min. Lung homogenates were stored at −20°C until analysis. Citrated blood was centrifuged at 664× *g*, plasma was frozen at −80°C. Before storage, all samples were filtered through a 22 µm filter (Millipore, Billerica, MA). Mouse s- and membrane-bound (m-)EPCR-levels were measured as described [38], using monoclonal rat-anti-mouse EPCR (Mab1560) as capture antibody and polyclonal goat-anti-mouse EPCR (GT262) as detection antibody. Interleukin (IL)-6, IL-10, IL-12p70, interferon (IFN)-γ and monocyte-chemoattractant protein (MCP)-1 were measured by cytometric bead array (CBA) multiplex assay (BD Biosciences, San Jose, CA) in accordance with the manufacturers' recommendations. Tumor necrosis factor-α (TNF-α; R&D systems, Minneapolis, MN), myeloperoxidase (MPO; HyCult-Biotechnology, Uden, The Netherlands) and TATc (Siemens Healthcare Diagnostics, Marburg, Germany) were measured with commercially available ELISA kits according to the manufacturers' instructions.

### Histology and immunohistochemistry

Paraffin-embedded 4 µm lung sections were stained with haematoxylin and eosin (H&E) and analyzed for inflammation and tissue damage, as described previously [33]–[35]. All slides were scored by a pathologist blinded for experimental groups for the following parameters: interstitial inflammation, necrosis, endothelialitis, bronchitis, edema, pleuritis, presence of thrombi and percentage of lung surface with pneumonia. All parameters were rated separately from 0 (condition absent) to 4 (most severe condition). The total histopathology score was expressed as the sum of the scores of the individual parameters, with a maximum of 32. Immunohistochemical detection of EPCR was performed as described [39]. Briefly, slides were incubated with a goat anti-mouse EPCR polyclonal antibody (GT262) as primary antibody, followed by incubation with rabbit-anti-goat IgG (SouthernBiotec, Birmingham, AL) as secondary antibody and polymer anti-rabbit-HRP (Immunologic, Duiven, The Netherlands) as tertiary antibody. Staining for granulocytes, using fluorescein isothiocyanate-labeled rat-anti-mouse Ly-6G mAb (BD Pharmingen, San Diego, CA) was performed as described previously [33], [34]. All slides were slightly counterstained with methylgreen. The total tissue area of the EPCR and Ly-6G stained slides was scanned with a slide scanner (Olympus dotSlide, Tokyo, Japan) and the obtained scans were exported in TIFF format for digital image analysis. The digital images were analyzed with ImageJ (version 2006.02.01, National Institutes of Health, Bethesda, MD) and the immunopositive (Ly6G+) area was expressed as the percentage of the total lung surface area.

### Statistical analysis

Patient data are expressed as dot plots with medians. Mouse data are expressed as box and whisker plots showing the smallest observation, lower quartile, median, upper quartile and largest observation or as medians with interquartile ranges. Comparisons between groups were conducted using the Mann-Whitney *U* test. For survival studies Kaplan-Meier analyses followed by log rank test were performed. All analyses were done using GraphPad Prism version 5.01 (GraphPad Software, San Diego, CA). *P*-values lower than 0.05 were considered statistically significant.

## Results

### Increased plasma sEPCR levels in patients with severe melioidosis

**Figure 1:**
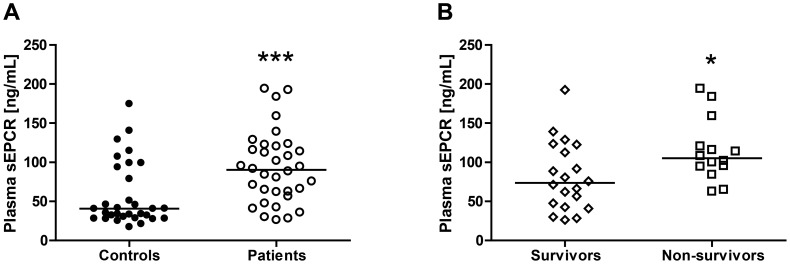
Plasma sEPCR levels of patients with culture proven melioidosis. Increased levels of sEPCR in plasma of patients (*n* = 34) with septic melioidosis compared to healthy controls (*n* = 32) (A). Increased levels of sEPCR in patients who died of melioidosis (*n* = 14) compared to patients that survived (*n* = 20) (B). Horizontal lines represent medians. ***P<0.001 for the difference between patients and controls; *P<0.05 for the difference between survivors (*n* = 20) and non-survivors (*n* = 14) (Mann-Whitney *U* test).

To obtain insight into EPCR expression and shedding during melioidosis, we measured sEPCR protein levels in plasma from 34 septic patients with culture proven *B. pseudomallei* infection and 32 local healthy controls. Fourteen (41%) patients with melioidosis died in-hospital. sEPCR was elevated in melioidosis patients with median plasma concentrations approximately 2.6-fold higher than in healthy subjects (Figure 1A; median 35 (28–83) ng/mL in controls versus 90 (61–121) ng/mL in patients, *P*<0.001). Moreover, sEPCR levels in non-survivors were significantly higher than in survivors (Figure 1B; survivors: median 74 (44–120) ng/mL *versus* non-survivors: 105 (92–130) ng/mL, *P*<0.05), indicating that high sEPCR levels are associated with poor outcome. In 8 patients in whom a follow-up sample was obtained, sEPCR levels remained unaltered (*P* = 0.38, data not shown).

### EPCR-expression is upregulated during murine melioidosis

**Figure 2:**
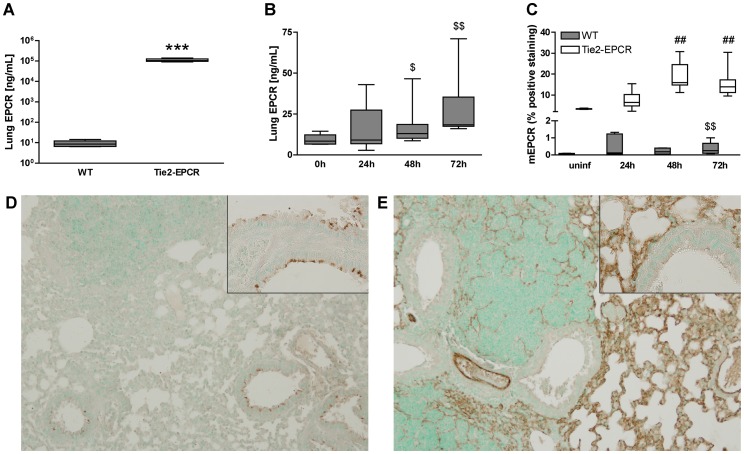
EPCR measurements in lungs of uninfected mice and mice infected with *B. pseudomallei*. Uninfected EPCR-overexpressing mice (Tie2-EPCR mice) have more than 10.000-fold higher levels of lung EPCR as measured by ELISA (A). Mice, inoculated with 300 CFU of *B. pseudomallei* and sacrificed after 24, 48 and 72 hours, had increased levels of EPCR in their lungs when compared to uninfected WT mice (B). Immunohistochemical staining of uninfected and *B. pseudomallei*-infected lung tissue showed increased EPCR-positive staining after 72 hours of infection in WT mice compared to uninfected controls and after 48 and 72 hours of infection in Tie2-EPCR mice when compared to uninfected Tie2-EPCR mice (C). Representative EPCR-staining of lung tissue of WT (D) and Tie2-EPCR (E) mice, 48 hours after infection (magnification 100×). Interestingly, at this time point WT mice showed EPCR-positive staining of bronchial epithelial cells (inset Figure D), whereas Tie2-EPCR mice (inset Figure E) did not (magnification 400×). Data are expressed as box-and-whisker plots showing the smallest observation, lower quartile, median, upper quartile and largest observation. Grey boxes represent WT mice, white boxes represent Tie2-EPCR mice (*n* = 8–9 mice per group for each time point). ***P<0.001 for WT versus Tie2-EPCR mice; ^$^ *P*<0.05 and ^$$^ *P*<0.01 for infected versus uninfected WT mice; ^##^ *P*<0.01 for infected versus uninfected Tie2-EPCR mice (Mann-Whitney *U* test).

A large part of severe melioidosis cases presents with pneumonia with bacterial dissemination to distant body sites [3], [5]. Considering that it is not feasible to study the role of EPCR at tissue level during human melioidosis, we used our well established mouse model in which mice are intranasally inoculated with live *B. pseudomallei* to induce pneumonia-derived melioidosis [33]–[35]. Mice were sacrificed after 24, 48 or 72 hours and total (s- and m-) EPCR protein levels were measured in lung homogenates of mice infected with *B. pseudomallei* and compared with uninfected mice. Figure 2A shows that in uninfected Tie2-EPCR mice median lung EPCR levels were more than 10.000-fold higher than in uninfected WT mice; *P*<0.0001). Infection of WT mice with *B. pseudomallei* was associated with a significant increase in EPCR levels in lung homogenates at 48 and 72 hours after inoculation (for both time points *P*<0.05 versus baseline; Figure 2B). Finally, we analyzed expression of mEPCR in lung tissue slides of WT mice and Tie2-EPCR mice during the course of the infection by immunohistochemistry and quantification by digital imaging. We compared these data with mEPCR-expression in uninfected mice. Infected WT mice had significantly higher lung mEPCR levels after 72 hours of infection when compared to their uninfected WT controls (*P*<0.05; Figure 2C). Infected Tie2-EPCR mice expressed higher levels of lung mEPCR after 48 and 72 hours (*P*<0.01; Figure 2C) when compared to uninfected Tie2-mice. Figure 2D (WT) and E (Tie2-EPCR) show representative photographs of mEPCR-stained lungs 48 hours after infection. Clearly, mEPCR was abundantly present in lung tissue of Tie2-EPCR mice showing mEPCR-positive staining not only in small arterioles but also in lung capillaries (Figure 2E). Interestingly, 48 hours after infection in WT mice mEPCR-positive staining was seen in bronchial epithelium (inset Figure 2D), while this was absent in bronchioles of Tie2-EPCR mice (inset Figure 2E). No evident mEPCR-positive staining was seen in lungs of uninfected WT mice (data not shown). Together, these data indicate that during murine infection with *B. pseudomallei* EPCR is upregulated, suggesting that this receptor is associated with the host defense against this pathogen.

### EPCR-overexpression facilitates pulmonary bacterial growth and dissemination to distant organs during experimental melioidosis

**Figure 3:**
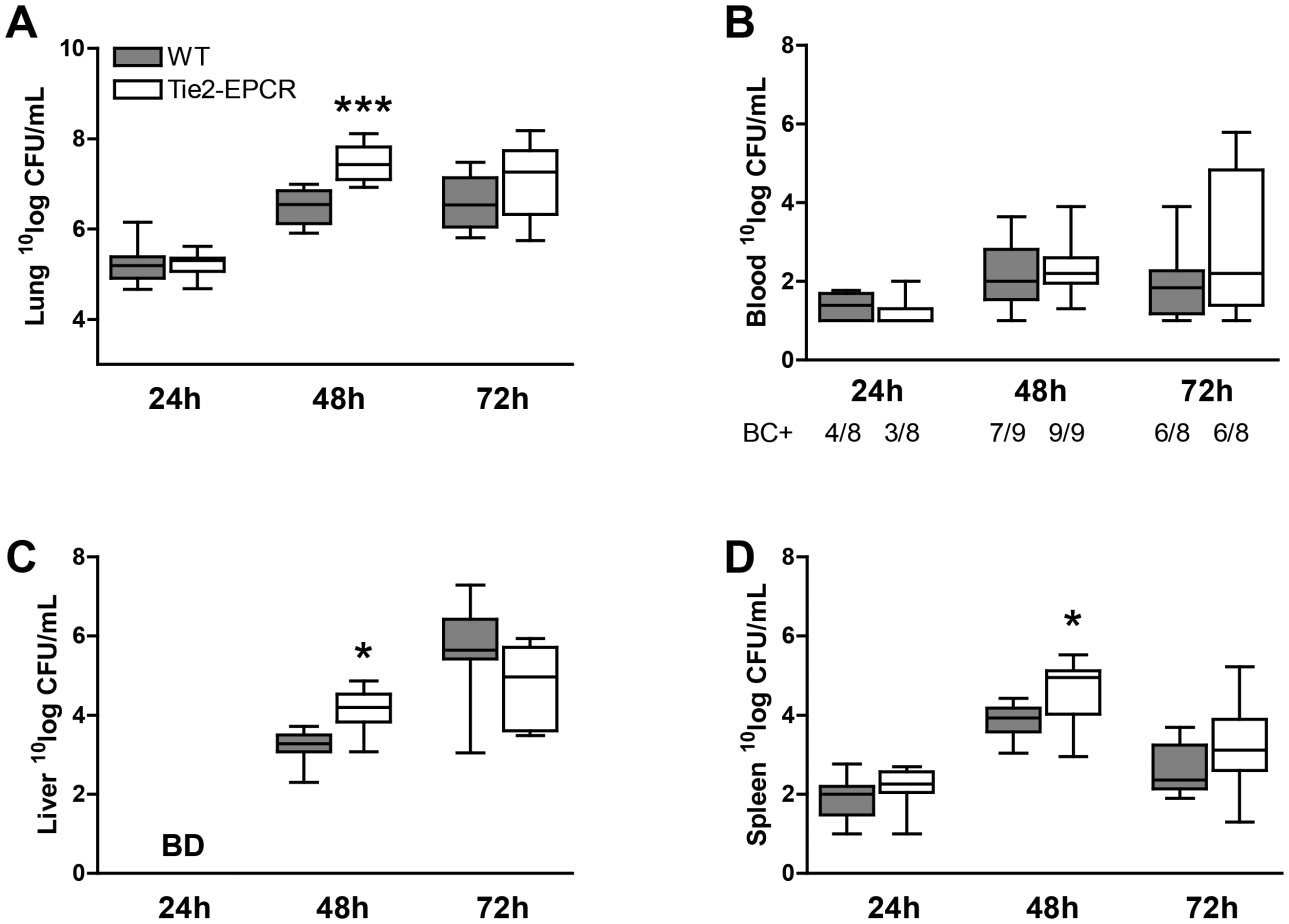
Overexpression of EPCR facilitates bacterial growth and dissemination. Mice were inoculated with 300 CFU of *B. pseudomallei* and sacrificed after 24, 48 and 72 hours. Bacterial loads were determined in lung homogenates (A), blood (B), liver homogenates (C) and spleen homogenates (D). Data are expressed as box and whisker plots showing the smallest observation, lower quartile, median, upper quartile and largest observation. Grey boxes represent WT mice, white boxes represent Tie2-EPCR mice (*n* = 8–9 mice per group for each time point). *P<0.05 and **P<0.01 for WT versus Tie2-EPCR mice (Mann-Whitney *U* test).

To investigate whether overexpression of EPCR impacts on local pulmonary bacterial growth, we determined bacterial loads in lung homogenates of WT mice 24, 48 and 72 hours after intranasal inoculation and compared them with bacterial loads in Tie2-EPCR mice. Forty-eight hours after inoculation with *B. pseudomallei* Tie2-EPCR mice had increased bacterial loads in their lungs when compared to WT mice (*P*<0.001; Figure 3A), while at 72 hours the differences were no longer evident. In order to determine whether EPCR-overexpression would impact on bacterial dissemination, we measured bacterial loads in blood, liver and spleen homogenates, 24, 48 and 72 hours after intranasal inoculation with *B. pseudomallei*. After 24 hours of infection almost 50% of all mice were bacteremic, while after 48 and 72 hours the majority (>75%) of mice had positive blood cultures (Figure 3B). However, no differences were seen at any of the time points in bacterial loads in blood between WT and Tie2-EPCR mice (Figure 3B). On the contrary, Tie2-EPCR mice displayed increased bacterial loads in liver and spleen homogenates 48 hours after infection (both *P*<0.05: Figure 3C–D), while at 72 hours no differences were seen anymore (Figure 3C–D). Thus, overexpression of EPCR facilitates pulmonary and systemic growth of *B. pseudomallei*.

### EPCR-overexpression is associated with increased lung inflammation

**Figure 4:**
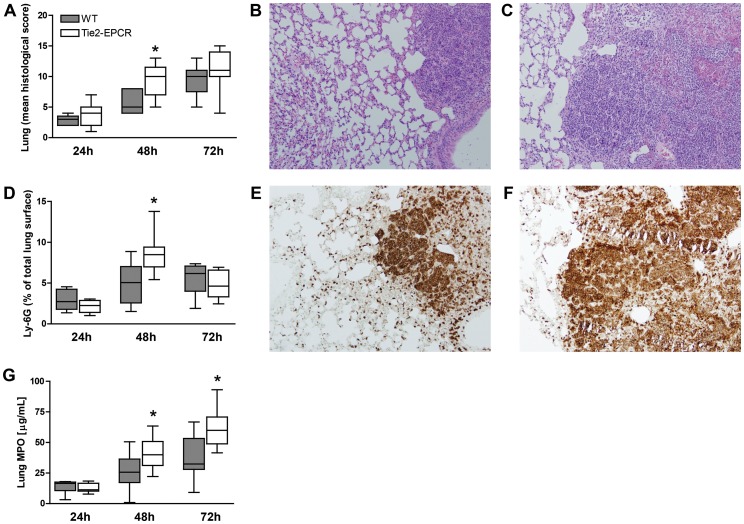
Lung pathology. Fourty-eight hours after infection Tie2-EPCR mice showed increased lung damage compared to WT mice as reflected by increased mean histological scores as described in the Methods section (A). Both WT (B) and Tie2-EPCR (C) mice infected with 300 CFU of *B. pseudomallei* showed inflammatory infiltrates in the lungs characterized by interstitial inflammation together with necrosis, endothelialitis, bronchitis, edema, thrombi and pleuritis 48 hours after inoculation (H&E staining ×100). Increased granulocyte migration in Tie2-EPCR mice infected with *B. pseudomallei* was seen 48 hours after infection (D). Representative photographs of Ly-6G-immunostaining (original magnification ×100) for granulocytes of WT (E) and Tie2-EPCR (F) mice showing significantly increased neutrophil influx at 48 h post-inoculation. Tie2-EPCR mice showed increased myeloperoxidase (MPO) levels compared to WT (G) in lung homogenates 48 and 72 h after infection. Data are expressed as box and whisker plots showing the smallest observation, lower quartile, median, upper quartile and largest observation. Numbers of granulocytes are expressed as the percentage of the total lung surface area. Grey boxes represent WT mice, white boxes represent Tie2-EPCR mice (*n* = 8–9 mice per group for each time point). *P<0.05 and **P<0.01 for WT versus Tie2-EPCR mice (Mann-Whitney *U* test).

Our murine model of melioidosis is associated with profound lung pathology [33]–[35]. In our study, both WT and Tie2-EPCR mice infected with *B. pseudomallei* showed inflammatory infiltrates in the lungs characterized by interstitial inflammation together with necrosis, endothelialitis, bronchitis, edema, thrombi and pleuritis (Figure 4A–C). The extent of lung inflammation, quantified by the semi-quantitative scoring system as described in the Methods section, was significantly greater in Tie2-EPCR mice at 48 hours after infection when compared to WT mice (*P*<0.05; Figure 4A). Figure 4B (WT mice) and 4C (Tie2-EPCR mice) show representative H&E-stainings of lung tissue 48 hours after intranasal inoculation with *B. pseudomallei*. As neutrophils play an important role in the host defense during melioidosis [3], [5], we next analysed neutrophil recruitment to lung tissue. Lung tissues were stained for Ly-6G, a marker for granulocytes, in order to quantify granulocyte influx. Clearly, 48 hours after infection Tie2-EPCR mice displayed significantly higher granulocyte counts in their lungs when compared to WT mice (*P*<0.05 at 48 hours post-infection; Figure 4D). Figure 4E (WT) and F (Tie2-EPCR) show representative photographs of Ly-6G-stainings of lung tissue 48 hours after intranasal inoculation with *B. pseudomallei*. These data showing enhanced neutrophil influx in Tie2-EPCR mice could be confirmed by elevated lung MPO concentrations, a marker for total neutrophil numbers, 48 and 72 hours after infection as compared to controls (Figure 4G). Altogether these data indicate that overexpression of EPCR results in enhanced lung inflammation and increased neutrophil influx during murine infection with *B. pseudomallei* starting from 48 hours after infection onwards.

### Induction of pro-inflammatory cytokines in EPCR-overexpressing mice

Since cytokines and chemokines are important regulators of the inflammatory response to *B. pseudomallei* [3]–[5], [40], we measured pulmonary and plasma levels of TNF-α, IL-6, IL-10, IL-12p70, IFN-γ and MCP-1 (Table 1). At 24 hours after inoculation all cytokine levels were low in both lung homogenates and plasma; whereas in lung homogenates no differences between mouse strains were seen at this early time point, in plasma significantly increased levels of TNF-α and IL-6 were detected in Tie2-EPCR mice as compared to WT mice (for TNF-α: 10 (5.9–14) *versus* 21 (11–22) pg/mL, *P*<0.05, for IL-6: 44 (13–65) *versus* 101 (73–161) pg/mL, *P*<0.001, data not shown). After 48 hours increases were found for almost all cytokines in lung homogenates, most prominent for TNF-α, IL-6 and MCP-1. These proinflammatory mediators were significantly more elevated in Tie2-EPCR mice when compared to WT mice (*P*<0.05, <0.001 and <0.01 for TNF-α, IL-6 and MCP-1 respectively; Table 1). Interestingly, IL-12p70 as well as IFN-γ levels, both Th1 cytokines, were decreased in lung homogenates of Tie2-EPCR mice 48 hours after infection (*P*<0.05 for both IL-12p70 and IFN-γ). Cytokine and chemokine levels in the lungs did not differ between groups at 72 hours after infection (data not shown). No significant differences were seen in plasma for any of the cytokines measured at the later time points.

**Table 1:** Cytokine concentrations in lung homogenates and plasma of WT and Tie2-EPCR mice during experimental melioidosis.

	WT	Tie2-EPCR
***pg/mL***	**Lung homogenates**	
**TNF-α**	1983 (1304–2739)	3141 (2533–3736)*
**IL-6**	2385 (1518–3294)	5902 (4080–9361)***
**IL-10**	7.8 (3.4–12)	15 (4.0–18)
**IL-12p70**	43 (28–48)	19 (10–26)*
**IFN-γ**	41 (28–62)	24 (20–29)*
**MCP-1**	5294 (4462–5933)	7971 (6573–8767)**
	**Plasma**	
**TNF-α**	23 (21–36)	37 (27–43)
**IL-6**	145 (91–400)	368 (170–816)
**IL-10**	BD	BD
**IL-12p70**	22 (14–28)	14 (5.9–22)
**IFN-γ**	490 (407–553)	405 (360–541)
**MCP-1**	245 (230–512)	363 (206–584)

Pulmonary and plasma cytokine levels 48 hours after intranasal infection with 300 CFU of *B. pseudomallei*. Wild type (WT) mice are compared with mice overexpressing EPCR (Tie2-EPCR mice). Data are expressed as median (interquartile ranges) of *n* = 8–9 mice per group per time point. BD below detection limits, IFN-γ interferon-γ, IL interleukin, MCP-1 monocyte-chemoattractant protein-1, TNF-α tumor necrosis factor-α.

* *P*<0.05,

** *P*<0.01 and

*** *P*<0.001 for WT versus Tie2-EPCR mice (Mann-Whitney *U* test).

### EPCR-overexpression hampers coagulation activation during infection with *B. pseudomallei*

**Figure 5:**
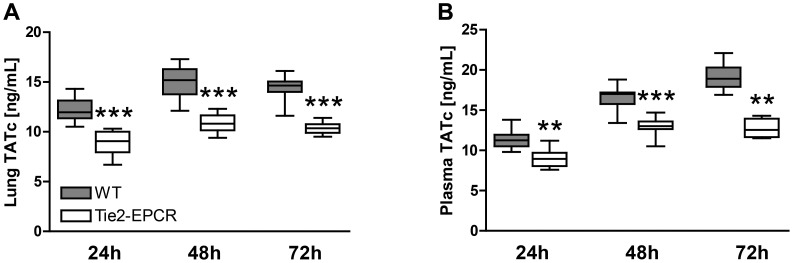
Tie2-EPCR mice show increased activation of coagulation both in lung homogenates and plasma. Mice were infected intranasally with 300 CFU of *B. pseudomallei*. After 24, 48 and 72 hours coagulation activation (TATc) was measured in lung homogenates (A) and plasma (B). At all time points decreased activation of coagulation was seen in Tie2-EPCR mice when compared to WT mice. Data are expressed as box and whisker plots showing the smallest observation, lower quartile, median, upper quartile and largest observation. Grey boxes represent WT mice, white boxes represent Tie2-EPCR mice (*n* = 8–9 mice per group for each time point). **P<0.01 and ***P<0.001 for WT versus Tie2-EPCR mice (Mann-Whitney *U* test).

Considering the important role for EPCR in the regulation of hemostasis [12], we next wondered whether EPCR overexpression would impact on activation of coagulation. Therefore TATc, a central parameter of coagulation-induction, was measured in lung homogenates and plasma of WT and Tie2-EPCR mice 24, 48 and 72 hours after inoculation with *B. pseudomallei*. At all time points, EPCR-overexpression hampered coagulation activation as reflected by decreased levels of TATc, both in lung homogenates and plasma, in Tie2-EPCR mice (*P*<0.01 - *P*<0.001 versus WT mice; Figure 5A–B).

### Deficiency of EPCR does not impact on bacterial growth and dissemination, pulmonary damage and neutrophil influx, but does induce coagulation activation in plasma

**Figure 6:**
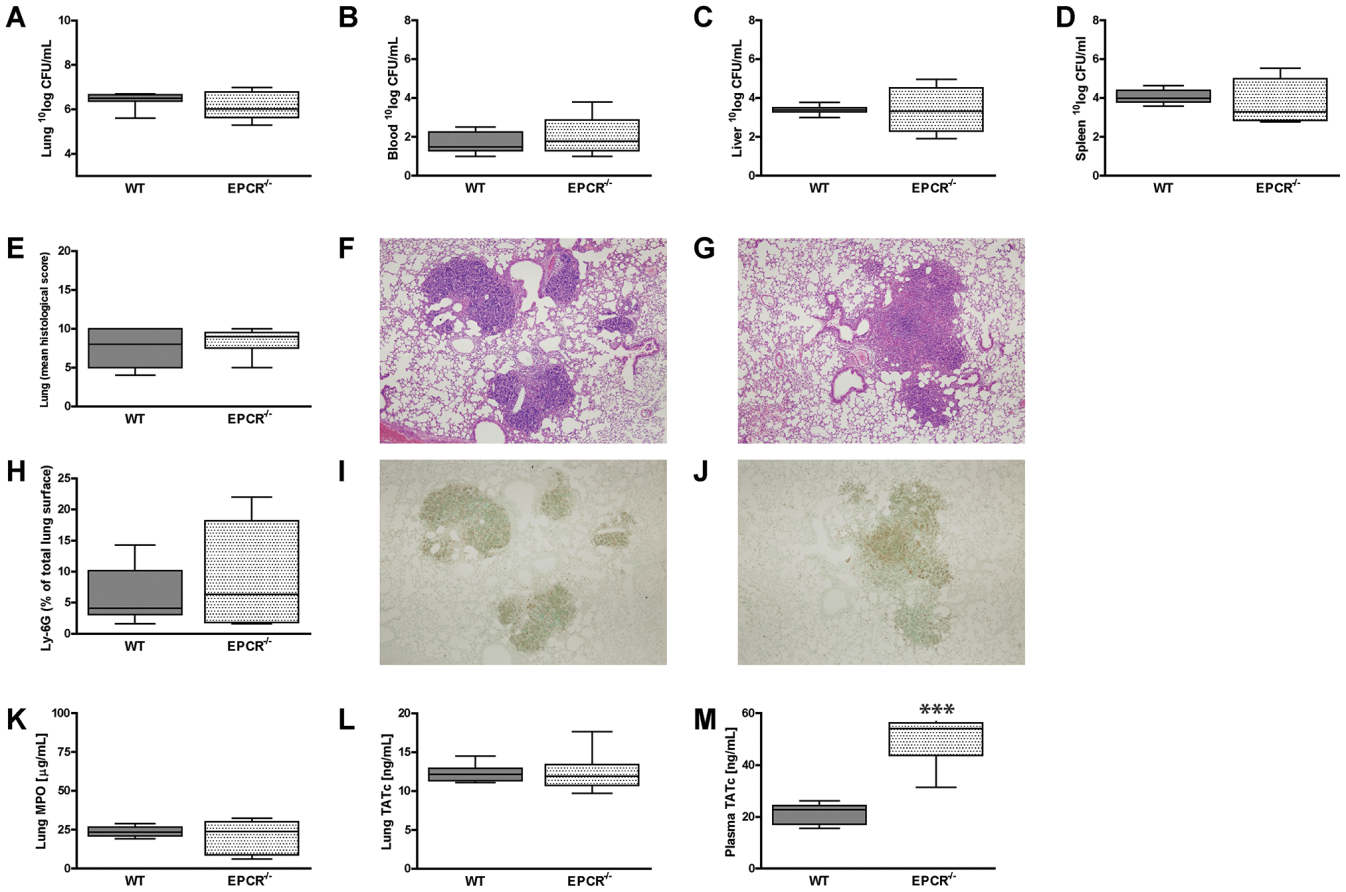
Deficiency of EPCR does not impact on bacterial growth and dissemination, pulmonary damage and neutrophil influx, but does induce coagulation activation in plasma. WT and EPCR-deficient (EPCR^−/−^) mice were inoculated with 300 CFU of *B. pseudomallei* and sacrificed after 48 hours. Bacterial loads were counted in lung homogenates (A), blood (B), liver homogenates (C) and spleen homogenates (D). Histopathology scores (E) were assessed as described in the Methods section. Figure F (WT) and G (EPCR^−/−^) show representative photographs of infected lung tissue 48 hours post-inoculation (H&E staining, original magnification ×100). Neutrophil influx was determined by Ly6G-staining of lung tissue (H). Figure I (WT) and J (EPCR^−/−^) show representative photographs of lung Ly6G-staining 48 hours post-inoculation (original magnification ×100). Levels of myeloperoxidase (MPO) were measured in lung homogenates (K). Activation of coagulation (TATc) was measured both in lung homogenates (L) and in plasma (M). Data are expressed as box and whisker plots showing the smallest observation, lower quartile, median, upper quartile and largest observation. Grey boxes represent WT mice, white boxes represent EPCR^−/−^ mice (*n* = 8–9 mice per group for each time point). *P<0.05 for WT versus EPCR^−/−^ mice (Mann-Whitney *U* test).

After having established the detrimental role for overexpression of EPCR during experimental melioidosis with respect to bacterial growth, lung pathology, neutrophil influx and cytokine production, we wondered whether deficiency of EPCR would have the opposite effect. Therefore, we infected WT and EPCR^−/−^ mice with 300 CFU of *B. pseudomallei* and sacrificed them after 48 hours, as at this time point the most prominent differences were seen in the experiments comparing WT and Tie2-EPCR mice. EPCR-deficiency did not impact on pulmonary bacterial loads (Figure 6A) or bacterial dissemination to blood (Figure 6B), liver (6C) or spleen (6D). In addition, EPCR^−/−^ mice neither showed any significant differences in lung damage reflected as the total sum of pathology scores (Figure 6E). Similarly, absence of EPCR did not impact on neutrophil influx into the lungs as indicated by percentages of Ly6G-staining in WT and EPCR^−/−^ mice (Figure 6H). Figure 6I (WT) and 6J (EPCR^−/−^ mice) show representative photographs of this Ly-6G-staining, 48 hours after infection. In accordance, no differences were seen between mouse strains in MPO levels (6K) either. Moreover, no differences between WT and EPCR^−/−^ mice were seen in cytokine and chemokine levels (TNF-α, IL-6, IL-10, IL-12p70, IFN-γ and MCP1), neither in lung homogenates, nor in plasma (data not shown). Finally, deficiency of EPCR did impact on coagulation in the systemic compartment. Whereas in lung homogenates no differences were seen between WT and EPCR^−/−^ mice (6L), in plasma EPCR^−/−^ mice displayed significantly increased activation of coagulation as reflected by higher levels of TATc (*P*<0.001; Figure 6M). Taken together these data indicate that although deficiency of EPCR leads to increased activation of coagulation in the circulation, it does not impact on bacterial growth, lung pathology, neutrophil influx or cytokine production during murine melioidosis.

## Discussion

In this study investigated the role of EPCR during melioidosis. We found that patients with culture-proven melioidosis have significantly increased levels of sEPCR in their plasma and that elevation of sEPCR was associated with higher mortality. Mice overexpressing EPCR showed increased bacterial growth and dissemination after infection with *B. pseudomallei* together with increased lung histopathology scores, increased influx of neutrophils in the lungs and decreased coagulation activation. On the contrary, after infection with *B. pseudomallei*, mice deficient for EPCR did not show any difference when compared to WT controls, except for enhanced coagulation activation in plasma 48 hours after infection. These data indicate that while endogenous EPCR does not impact on the host response during Gram-negative pneumonia-derived sepsis caused by *B. pseudomallei* to a significant extent, transgenic overexpression of this receptor impairs outcome.

Clinical and preclinical studies have provided evidence for a role for EPCR during sepsis [19]–[23], [41]. It has been hypothesised that sepsis results in down-regulation of mEPCR levels on injured endothelium, which consequently may impair the ability to generate APC. Indeed, in vitro studies with endothelial cells have shown that proinflammatory cytokines including TNF-α [42]–[44] and IL-1β [45], [46] are able to down-regulate mEPCR. Furthermore, in children with meningococcal sepsis endothelial mEPCR levels were lower than those in controls [28]. Down-regulation of mEPCR may occur both via inhibition of EPCR gene transcription [42], [44] and via protease-mediated shedding from the endothelial cell surface [18], releasing the soluble form of EPCR in the circulation. In accordance, several studies have demonstrated increased levels of plasma sEPCR during sepsis in general [25], [26] or in Gram-negative sepsis only [27]. Our data demonstrating increased sEPCR levels during melioidosis are in fully in line with this. On the other hand, some studies showed unchanged [28] or decreased sEPCR levels [29] during sepsis. This discrepancy can possibly be explained by different ELISA methods used to measure sEPCR [29] and/or the frequency of the A3-haplotype of sEPCR in the study population [41]. Indeed, carriers of the A3-haplotype have significantly higher sEPCR levels than non-carriers and a variation in the percentage of A3-carriers in the study population may influence the final results, as was revealed earlier [29]. Furthermore, we demonstrated that increased levels of sEPCR are correlated with a poor survival in patients during sepsis caused by *B. pseudomallei*, which is in line with earlier observations [27]; however, others failed to show a similar association [25]. We also could not detect any changes in sEPCR levels during the hospital stay. Altogether our data on sEPCR in human plasma samples might indicate a role for sEPCR during human melioidosis, but could also reflect a coincidental ‘bystander’ effect. Evidently, more research has to be done, especially on the expression of EPCR on freshly harvested inflammatory cells such as neutrophils, monocytes and macrophages during human melioidosis.

To further investigate the role of EPCR during melioidosis we used mice overexpressing this receptor and infected them with *B. pseudomallei*. Infection resulted in an increase in total EPCR levels in whole lung homogenates, indicative of the sum of m- and sEPCR in the pulmonary compartment. Staining for mEPCR (expressed as percentage positive staining of lung tissue quantified by digital imaging) in WT mice showed clear differences in EPCR levels between uninfected and infected lungs, suggesting that the increase in total EPCR concentrations was caused by both shedding of sEPCR and upregulation of mEPCR. Of note, however, infected WT mice displayed increased mEPCR-expression on the luminal side of the bronchial epithelium, which was absent in bronchioli of infected Tie2-EPCR mice. This mEPCR upregulation was restricted to the bronchial epithelium, as still little mEPCR-staining was seen in other tissues, especially not the endothelium. Previous studies have documented constitutional EPCR expression by respiratory epithelial cells [47], [48]. The enhanced epithelial EPCR expression observed here is remarkable in light of earlier studies showing that endothelial mEPCR expression is down-regulated by proinflammatory cytokines including TNF-α and IL-1β [42]–[46]. The fact that Tie2-EPCR mice did not show increased epithelial mEPCR expression could be related to the altered proinflammatory milieu in these animals and/or to constitutively high endothelial cell mEPCR levels. Possibly, due to already strongly enhanced mEPCR expression in endothelial tissues in Tie2-EPCR mice, the bronchial epithelium was less triggered to express mEPCR in reaction to pro-inflammatory stimuli or endothelial EPCR expression might even have counterregulated EPCR expression in these Tie2-EPCR mice. In our model this is of main importance as the lungs were the primary focus of disease, and possibly, the downregulation of EPCR on bronchial epithelium in Tie2-EPCR mice might have contributed to the detrimental phenotype.

Transgenic overexpression of EPCR in mice resulted in impaired outcome during experimental murine melioidosis, as indicated by increased bacterial loads at the primary site of infection and distant organs and increased tissue damage. Our data contrast with previous studies showing that EPCR-overexpression protects mice from a lethal dose of LPS [23] and from ventilator-induced lung injury (VILI) caused by high tidal volume ventilation [49]. Obviously, the behaviour of Tie2-EPCR mice depends on the experimental trigger given. In this view it is important to realize that in our model we used a living and replicating bacterium, *B. pseudomallei*, as inflammatory trigger, implying a continuous and increasing proinflammatory stimulus, which is different from LPS or non-infectious stimuli such as VILI. Tie2-EPCR mice showed decreased coagulation activation during experimental melioidosis, as reflected by decreased TATc levels, which corresponds with earlier observations in these animals during VILI [49] and after FXa administration [23]. Moreover, Tie2-EPCR mice have higher circulating levels of APC [23] and preliminary data from our laboratory indicate that transgenic overexpression of APC in mice [50] results in a phenotype during experimental melioidosis that resembles that of Tie2-EPCR mice, i.e. with enhanced bacterial growth and dissemination, and increased inflammation and injury. Together, these data suggest that an enhanced anticoagulant environment impairs infection control and containment in this model of pneumonia-derived sepsis. Of note, it is important to remark that it needs caution to simply extrapolate our data obtained from mouse experiments to the human situation. Recent data showed that genomic responses in mouse models poorly mimic human inflammatory diseases, including endotoxemia challenge [51]. In murine models, the responses on proinflammatory stimuli may be obviously dependent on factors such as the mouse strain used, the route of challenge and the bacterial strain used. Equally these factors may vary in humans, who, in addition are typically older, have co-morbidities that may modify the course of the disease and have received treatment in the form of fluids and antibiotics.

Besides its effects on coagulation, overexpression of EPCR is expected to result in cytoprotective and anti-inflammatory effects via binding of APC or FVIIa to EPCR and subsequent signalling via PAR1 [9], [15]. In contrast, Tie2-EPCR mice displayed a proinflammatory phenotype particularly at 48 hours after infection, most likely caused by the increased bacterial loads; with the readouts used here anti-inflammatory effects could not be detected, leaving the mechanism by which overexpression of EPCR disturbs host defense mechanisms open for discussion. Nonetheless, our results demonstrate that high EPCR levels do not prevent excessive inflammation or death during melioidosis.

Cytokines play an important role in the host defense against *B. pseudomallei* [3], [4], [40]. Melioidosis patients demonstrated elevated serum levels of TNF-α, IL-12 and IFN-γ in recent clinical studies [40], [52]. The pro-inflammatory cytokine IFN-γ is produced by cytotoxic T-cells and natural killer cells and has an important protective role in early resistance against *B. pseudomallei* infection. Earlier evidence showed that blockage of IFN-γ lowered the LD50 from >5*10^5^ CFU to ∼2 CFU and was associated with marked increases in bacterial loads in various organs [53]. In line, inhibition of IL-12, one of the predominant inducers of IFN-γ, resulted in increased mortality in the same model [53]. The pro-inflammatory cytokine TNF-α is also likely to be an important element of the early immune response to *B. pseudomallei* as passive immunization against this mainly macrophage derived-cytokine enhances lethality in experimental murine melioidosis [53]. We therefore measured levels of IFN- γ, IL-12p70 and TNF- α in our model. Our results showed marked increases in all of these three cytokines in EPCR-overexpressing mice, demonstrating that EPCR elicits a more profound host response during experimental melioidosis.

EPCR-deficient mice did not have a clear phenotype upon infection with *B. pseudomallei*. We chose the 48-hour time point as this was most discriminative in the studies using Tie2-EPCR and WT mice. Our data are in contrast with earlier data showing that, upon LPS challenge, mice fully deficient for EPCR exhibited more cytokine generation, neutrophil sequestration in the lungs and a higher mortality rate than mice heterozygously deficient for EPCR [22]. Also, mice with a severe EPCR-deficiency displayed hemodynamic and cardiac alterations and a lower survival after LPS administration [54]. Similarly, EPCR-blocking antibodies exacerbated the inflammatory response to bacterial sepsis in baboons [20], [21]. Dissimilarities in the models used may at least in part explain these differences: whereas in the earlier studies [20]–[22], [54] the challenges used resulted in a brisk systemic inflammatory response syndrome, the current model of melioidosis is associated with a growing bacterial load in the lung with subsequent dissemination and inflammation to distant body sites. EPCR-deficient mice did have increased coagulation activation during meliodosis, which is in line with earlier findings showing that EPCR-deficient mice developed more and larger thrombi upon FXa- or LPS-challenge than control mice [22], [37].

The current study is the first to report on the role of EPCR in melioidosis. We showed that in human melioidosis plasma sEPCR levels are elevated and associated with increased mortality. Murine studies showed that in melioidosis overexpression of EPCR is detrimental, with respect to bacterial growth and local and systemic inflammatory responses, while EPCR deficiency did not influence host responses. Together with previous studies from our laboratory [33], [34] these data point to an important role for mediators involved in hemostasis in the host defense against *B. pseudomallei*.
